# Comparison of diagnostic accuracy of screening tests ALT and ultrasound for pediatric non-alcoholic fatty liver disease

**DOI:** 10.1007/s00431-019-03362-3

**Published:** 2019-03-22

**Authors:** Laura G. Draijer, Sana Feddouli, Anneloes E. Bohte, Olga vd Baan Slootweg, Tammo H. Pels Rijcken, Marc A. Benninga, Jaap Stoker, Bart G. P. Koot

**Affiliations:** 1 Department of Pediatric Gastroenterology and Nutrition, Amsterdam University Medical Centers, Location Academic Medical Center/Emma Children’s Hospital, Meibergdreef 9, 1100 AZ Amsterdam, The Netherlands; 2 Department of Radiology and Nuclear Medicine, Amsterdam University Medical Centers, Location Academic Medical Center, Amsterdam, The Netherlands; 3 Obesity Clinic Heideheuvel, Soestdijkerstraatweg 129, 1213 VX Hilversum, The Netherlands; 4 Department of Radiology, Tergooi Hospital, Van Riebeeckweg 212, 1213 XZ Hilversum, The Netherlands

**Keywords:** NAFLD, Children, Obesity, Screening, ALT, Ultrasound

## Abstract

Alanine aminotransferase (ALT) and ultrasound (US) are the most commonly used tools for detecting non-alcoholic fatty liver disease (NAFLD). No direct comparison of these two modalities in children exists. We aimed to compare head-to-head the diagnostic accuracy of ALT and US and their combination for detecting NAFLD in children with obesity. Ninety-nine children with severe obesity underwent simultaneous serum-ALT and abdominal ultrasound (US steatosis score 0–3). Proton magnetic resonance spectroscopy was used as reference standard for detecting steatosis/NAFLD. ROC curve analyses were performed to determine diagnostic performance and to determine optimum screening cut-points aiming for a specificity ≥ 80%. The area under the ROC (AUROC) of ALT and US were not significantly different (0.74 and 0.70, respectively). At the optimal ALT threshold (≥40 IU/L), sensitivity was 44% and specificity was 89%. At the optimal US steatosis score (≥ 2), sensitivity was 51% and specificity was 80%. Combining ALT and US did not result in better accuracy than ALT or US alone.

*Conclusion*: ALT and US have comparable and only moderate diagnostic accuracy for detecting hepatic steatosis in children with obesity. A stepwise screening strategy combining both methods does not improve diagnostic accuracy.

**Table:** 

**What is Known:** • *Alanine aminotransferase* (*ALT*) *and ultrasound* (*US*) *are the most commonly used tools for detecting non-alcoholic fatty liver disease* (*NAFLD*)*.* *• ALT and ultrasound have mediocre accuracy in detecting steatosis in children with obesity.*	
**What is New:** *• In a head-to-head comparison, the difference in diagnostic accuracy of ALT and ultrasound in detecting steatosis is not significant.* *• A stepwise screening strategy combining both methods does not improve diagnostic accuracy*.

## Introduction

Non-alcoholic fatty liver disease (NAFLD) is well established as one of the complications of obesity. NAFLD includes different stages of disease: simple steatosis, steatohepatitis, fibrosis, and cirrhosis. The reported prevalence of NAFLD in children is 7.6% in general population studies and 38% in studies based on child obesity clinics [1–3]. Concomitant with the rise of obesity, the prevalence of NAFLD has increased during the last two decades, making NAFLD the most common chronic liver disease in children in the industrialized world [2, 4].

It is important to identify patients with obesity and NAFLD since advanced fibrosis is reported in up to 17% of children referred to liver centers after screening [4, 5]. In view of their long life expectancy, patients with significant fibrosis at pediatric age are particularly at risk of long-term complications during their lifetime, i.e., cirrhosis, liver failure, and hepatocellular carcinoma. In addition, NAFLD is also an independent risk factor for type 2 diabetes and, although still disputed, probably also for cardiovascular disease at adult age [6, 7].

Because of these long-term health risks and lack of symptoms until advanced disease occurs, screening for NAFLD in children with obesity is propagated in most major national and international obesity and hepatology guidelines [8]. The first step in screening is usually to detect steatosis, and subsequently, those patients identified are further evaluated for inflammation and fibrosis. Guidelines differ in their advice on the primary screening tool, alanine aminotransferase (ALT), and ultrasound (US). This disparity is due to the lack of data on the optimal primary screening tool to detect NAFLD.

Although the use of ALT and US as screening tools have been evaluated in previous studies, no study compares head-to-head the diagnostic accuracy of ALT and US [9–15]. Also, a stepwise screening approach that combines both tests has not yet been studied. The aim of the present study was to compare head-to-head the accuracy of ALT and US as screening tools for NAFLD in children with severe obesity. In addition, we evaluated whether a stepwise screening approach combining both tests increases the accuracy of screening.

## Methods

### Study design and patients

This was a cross-sectional study. Participants were recruited from the obesity clinic “Heideheuvel” in Hilversum, the Netherlands, during a 2-year study period between February 2008 and October 2010. Admission criteria for this program were severe obesity (body mass index *z*-score > 2.6) or obesity (BMI *z*-score > 2) along with obesity-related co-morbidity (dyslipidemia and hypertension). All children admitted to the obesity clinic between 8 and 18 years old were eligible for participation in our study. Exclusion criteria were known liver disease other than NAFLD (viral/autoimmune hepatitis, Wilson’s disease, hemochromatosis, alpha-1 antitrypsin deficiency), metabolic disease (b-oxidation defects, urea cycle defects), (history of) use of steatogenic medication, alcohol consumption >7 units/week, jejunoileal surgery, type 1 or type 2 diabetes mellitus, history of parenteral feeding, and contraindications for MRI. All participants underwent an ultrasound of the liver, venipuncture and a proton magnetic resonance spectroscopy (^1^H-MRS) of the liver before starting the lifestyle program at the obesity clinic within a time interval of 1 month. Accuracy data on ultrasound of a part of this study population were previously published by Bohte et al. [16]. The study was approved by the Medical Ethics Committee of the Academic Medical Center of the University of Amsterdam. Written informed consent was obtained from the participants and/or their legal guardians. We followed the Standards for Reporting of Diagnostic Accuracy (STARD) guidelines in this study on the accuracy of ALT and US in detecting hepatic steatosis [17].

### Clinical assessment

All measurements were performed at the start of the lifestyle program at the obesity clinic. Weight and length were measured to calculate the BMI *z*-score. The BMI *z*-score is the number of standard deviations (SD) from the mean on a standard BMI curve for age and gender. Children with a BMI *z*-score of > 2 (95th percentile) are considered obese, and those with a BMI *z*-score of > 2.6 (99th percentile) are considered severely obese [18, 19].

### Laboratory tests

Venous blood was sampled after an overnight fast for serum biochemistry studies. ALT, aspartate aminotransferase (AST), gamma-glutamyltransferase (γGT) and lipids were measured directly after blood sampling using standard laboratory methods by certified laboratory staff. Fasted insulin and glucose were used to calculate the homeostatic model assessment of insulin resistance score (HOMA-IR) [20]. An oral glucose tolerance test was performed to exclude the presence of type 2 diabetes*.* Hepatitis B and C, autoimmune hepatitis, alpha-1 antitrypsin deficiency, abetalipoproteinemia, hemochromatosis, and Wilson’s disease were excluded using the appropriate diagnostic tests.

### Index test 1: Alanine transaminase

We determined the accuracy of ALT at cutoffs commonly applied in screening guidelines: the sex-specific upper limit of normal (ULN) for healthy children (22 IU/L for girls and 26 IU/L for boys) [21]; 2× the sex-specific ULN (44 IU/L for girls and 52 IU/L for boys) [22]; and the cutoff mostly used by laboratories and physicians in clinical practice for both sexes (ALT ≥40 IU/L).

### Index test 2: Ultrasound

For this study, we used US systems (ATL HDI 5000, HD11, and IU22; Philips Healthcare, Best, the Netherlands) with 2–5- and 3.75-MHz curved-array transducers. The “abdominal general” setting was used on the US system for all the US examinations. Three radiologists with 5–20 years’ experience and more than 600 liver US examinations per year, performed and interpreted the US examination of all participants. All three radiologists were blinded for the data of this study. The following acknowledged scoring items were used: (1) echogenicity of liver parenchyma; (2) visualization of the diaphragm; (3) visualization of intrahepatic vessels; and (4) visualization of posterior part of the right hepatic lobe. Subsequently, the degree of liver steatosis was scored (Table 1). The “ultrasound steatosis score” (US score) was defined as the average score of the four items [23]. We determined the accuracy of US using each US score. As previously published, the interobserver agreement between the three radiologists was moderate to good (kappa 0.58 to 0.68) and the intraobserver agreement (kappa 0.82 to 0.91) was excellent.

**Table 1:** Scoring of hepatic steatosis with ultrasound (US score)

Score 0 = normal	Normal echogenicity of liver parenchyma. Normal visualization of the diaphragm and intrahepatic blood vessels.
Score 1 = mild steatosis	Slightly increased echogenicity of liver parenchyma. Normal visualization of the diaphragm and intrahepatic blood vessels.
Score 2 = moderate steatosis	Markedly increased echogenicity of liver parenchyma. Slightly decreased visualization of the diaphragm and intrahepatic blood vessels.
Score 3 = severe steatosis	Severely increased echogenicity of liver parenchyma. No or severely decreased visualization of the diaphragm, intrahepatic blood vessels, and posterior part of the right liver lobe.

*US score* ultrasound steatosis score

### Reference test: Proton magnetic resonance spectroscopy

^1^H-MRS spectra were acquired using a point-resolved spectroscopy sequence (TE/TR = 38/2000 ms) in a voxel of 20 × 20 × 20 mm^3^ during free breathing on a 3.0 Tesla MR system (Philips Healthcare, Best, the Netherlands). An open bore 1.0 Tesla MR scanner (Philips Healthcare, Best, the Netherlands) was used in participants with body weight more than 150 kg or an abdominal circumference of more than 150 cm. In this study, the presence of steatosis is defined as a liver fat percentage of > 1.8% measured by ^1^H-MRS. This cutoff has been validated to correspond with > 5% fat containing hepatocytes on liver histology, i.e., the histological definition of NAFLD [24]. ^1^H-MRS spectra were analyzed by a research fellow with 3 years of experience and supervised by an MR physicist with 8 years of experience. Both were blinded for the results of ALT and US. ^1^H-MRS is an accurate non-invasive tool to diagnose or exclude NAFLD [25].

### Statistical analysis

Patients’ demographic data, laboratory and imaging data were summarized with mean and standard deviation for continuous variables with a normal distribution or median and interquartile range (IQR) for continuous variables with a non-normal distribution. Receiver operating characteristic (ROC) analysis was used to evaluate the discriminatory power of the two screening tests. We compared the areas under the ROC curves (AUROC) in MedCalc by using a (pairwise) comparison according to the method used by Hanley and McNeil [26]. A value of *p* < 0.05 was considered a statistically significant difference. In addition, at the aforementioned ALT cutoffs and for all US scores, the diagnostic accuracy was calculated, including sensitivity, specificity, positive predictive value (PPV), negative predictive value (NPV), and positive and negative likelihood ratios (LR+, LR−) with 95% confidence intervals.

Subsequently, ALT and US were combined in a stepwise screening approach using the previously calculated optimal cutoffs of both tools. Diagnostic accuracy of those algorithms was calculated, including total accuracy in order to assess overall performance. The total accuracy is defined as (prevalence × sensitivity)/((1 − prevalence) × specificity) [27].

Since NAFLD has a high prevalence among children with obesity and missing the diagnosis has no direct harmful health effect, it is rational to primarily aim for an adequate specificity to limit the number of false positives and thereby avoid unnecessary distress among patients and a huge burden of unnecessary additional testing. In this study, we defined an adequate specificity as > 80%.

All analyses were performed with PASW Statistics 18 SPSS Inc., Chicago, IL; MedCalc Software; and with Microsoft Office Excel, Microsoft, Redmond, WA.

## Results

### Participants

In this study, 121 participants were consecutively included. Two participants withdrew from the study and the data on US and ^1^H-MRS were incomplete in 20 participants. In total, data of 99 children (42 male, 57 female) were analyzed. The mean age was 14.1 ± 2.1 years (range 8.3–17.8). The mean BMI *z*-score was 3.3 ± 0.3 SD (range 2.5–4.2). The baseline characteristics are summarized in Table 2*.* Hepatic steatosis was present in 43/99 children (43.4%).

**Table 2:** Baseline characteristics

Demographic	
Age (years)	14.1 (2.1)
Female, *n* (%)	57 (57.6)
Clinical	
Steatosis, *n* (%)	43 (43.4)
BMI *z*-score	3.3 (0.3)
Waist circumference, cm (IQR)	102 (14)
Biological data	
ALT, IU/L (IQR)	27 (22)
γGT, IU/L (IQR)	21 (9)
HOMA-IR (IQR)	3.17 (2.5)
Triglycerides, mmol/L (IQR)	0.85 (0.60)
Total cholesterol, mmol/L	4.02 (0.79)
HDL-cholesterol, mmol/L	1.07 (0.23)
LDL-cholesterol, mmol/L	2.52 (0.69)
Imaging data	
US steatosis score 0, *n* (%)	23 (23.2)
US steatosis score 1, *n* (%)	42 (42.4)
US steatosis score 2, *n* (%)	25 (25.3)
US steatosis score 3, *n* (%)	9 (9.1)

Continuous variables are expressed in mean with standard deviation in parentheses or median with interquartile range (IQR) in parentheses or *n* (%). *BMI* body mass index, *ALT* alanine aminotransferase, *AST* aspartate aminotransferase, *γGT* gamma-glutamyltransferase, *HOMA-IR* homeostatic model assessment of insulin resistance, *HDL* high-density lipoprotein, *LDL* low-density lipoprotein, *US* ultrasound

### Diagnostic accuracy

**Fig. 1:**
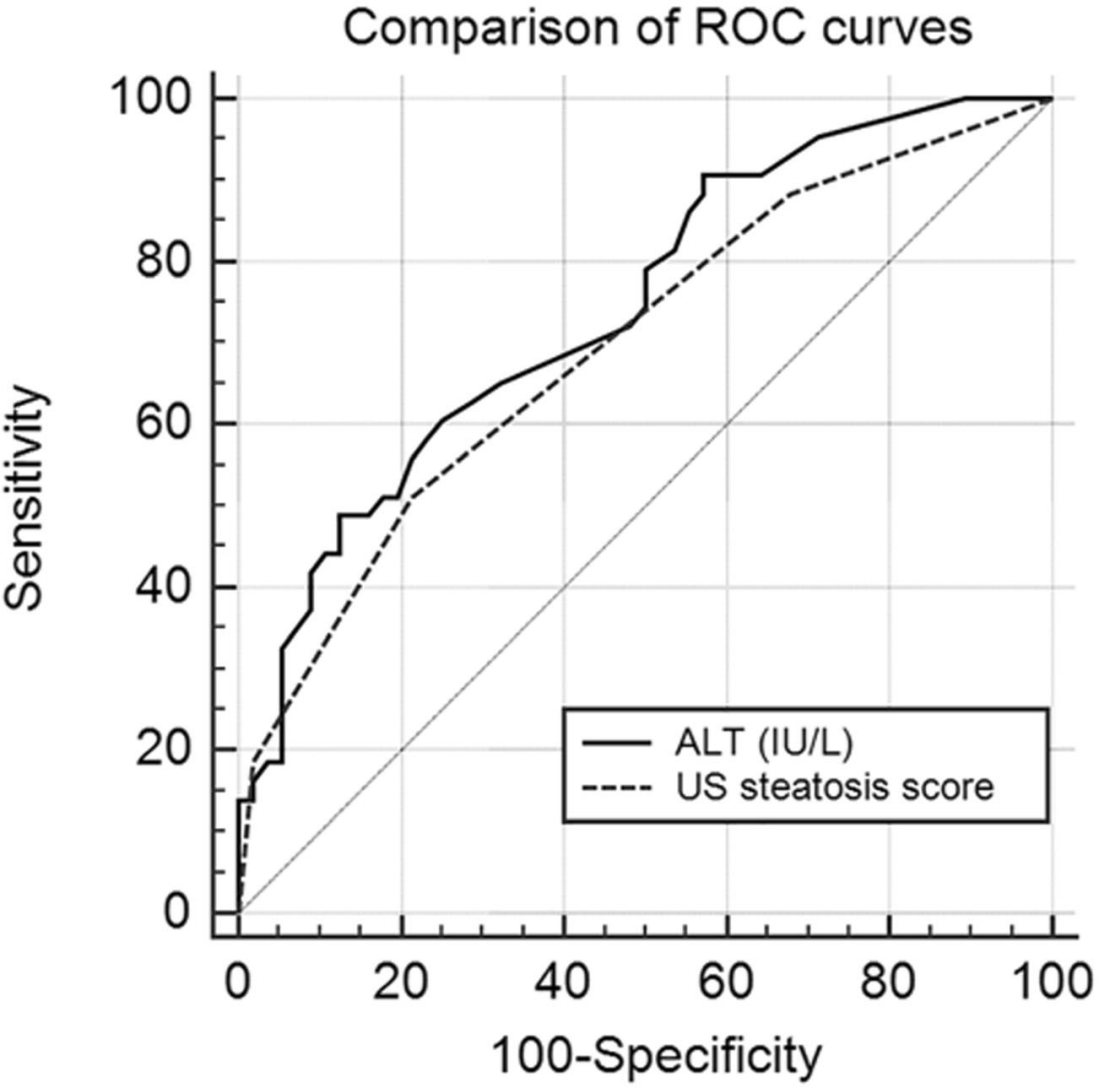
ROC curves of ALT and US. The AUROC of ALT and US were 0.70 (95% CI 0.60–0.79) and 0.74 (95% CI 0.65–0.83), respectively (NS). ROC, receiver operating curve; ALT, alanine aminotransferase; US, ultrasound; AUROC, area under the ROC curve

The ROC curves of ALT and US for detection of hepatic steatosis are presented in Fig. 1**.** The AUROC values of ALT and US were comparable: 0.74 (95% CI 0.65–0.83) and 0.70 (95% CI 0.60–0.79), respectively (*p* = 0.41).

#### ALT

The diagnostic accuracy of ALT at the aforementioned cutoffs is presented in Table 3. At a threshold of ALT ≥40 IU/L, the predetermined minimal specificity of 80% was reached, however, with a sensitivity of only 44%.

**Table 3:** Diagnostic accuracy of alanine aminotransferase (ALT)

Test (IU/L)	Sensitivity % (95% CI)	Specificity % (95% CI)	PPV % (95% CI)	NPV % (95% CI)	LR+ (95% CI)	LR− (95% CI)
ALT ≥ ULN *n* = 62	79 (64–90)	50 (36–64)	55 (47–62)	76 (62–85)	1.6 (1.2–2.1)	0.4 (0.2–0.8)
ALT ≥ 40 *n* = 25	44 (29–60)	89 (78–96)	76 (58–88)	68 (61–73)	4.1 (1.8–9.4)	0.6 (0.5–0.8)
ALT ≥ 2× ULN *n* = 13	23 (12–39)	95 (85–99)	77 (49–92)	62 (57–66)	4.3 (1.3–14.8)	0.8 (0.7–1.0)

ULN = 22 IU/L for girls, 26 IU/L for boys

*ALT* alanine aminotransferase, *ULN* upper limit of normal, *PPV* positive predictive value, *NPV* negative predictive value, *LR+* positive likelihood ratio, *LR−* negative likelihood ratio

#### Ultrasound

The diagnostic accuracy of US at different US scores is presented in Table 4. A US score ≥ 2 reached the predetermined minimal specificity of > 80%, however, with a sensitivity of only 51%.

**Table 4:** Diagnostic accuracy of ultrasound (US)

Test	Sensitivity % (95% CI)	Specificity % (95% CI)	PPV % (95% CI)	NPV % (95% CI)	LR+ (95% CI)	LR− (95% CI)
US ≥ 1 *n = 76*	88 (75–96)	32 (20–46)	50 (44–55)	78 (59–90)	1.3 (1.1–1.6)	0.4 (0.2–0.9)
US ≥ 2 *n = 34*	51 (35–67)	80 (66–88)	65 (51–77)	68 (60–75)	2.4 (1.3–4.3)	0.6 (0.4–0.9)
US ≥ 3 *n = 9*	19 (8–33)	98 (90–100)	89 (51–98)	61 (58–64)	10.4 (1.4–80.1)	0.8 (0.7–1.0)

*US* ultrasound steatosis score, *PPV* positive predictive value, *NPV* negative predictive value, *LR+* positive likelihood ratio, *LR−* negative likelihood ratio

#### Stepwise screening strategy

Different stepwise approaches combining ALT and US were evaluated. In the first algorithm, all patients with ALT ≥ 40 IU/L undergo additional US to increase the specificity. Only those with ALT ≥ 40 IU/L *plus* US score ≥ 2 were considered to have a positive screening result. In the second algorithm, to increase the sensitivity, US is performed in all patients with ALT < 40 IU/L. Patients with ALT ≥ 40 IU/L and those with ALT < 40/IU/L *plus* US score ≥ 2 were considered to have a positive screening result in this algorithm. Thirdly, to limit the number of additional ultrasounds, we determined if performing ultrasound in the group of patients with ALT ≥ ULN but < 40 IU/L increases the accuracy. Patients with ALT levels ≥ 40 IU/L and those with ALT ≥ ULN but < 40 IU/L *plus* US score ≥ 2 were considered to have a positive screening result. In the last algorithm, to increase the specificity, ultrasound is performed in all patients with ALT > ULN (Table 5*)*. A combination of ALT ≥40 IU/L and US score ≥ 2 increases the specificity of screening to 98% but decreases the sensitivity to 32%. The overall accuracy of this algorithm was 70%, which is comparable to the overall accuracy of ALT ≥40 IU/L alone (overall accuracy of 70%). All other algorithms also had a comparable and only mediocre overall accuracy. We also performed additional analyses assessing the algorithms using US as a first test, followed by ALT measurement. These combinations did not improve the accuracy of screening (data not shown).

**Table 5:** Diagnostic accuracy characteristics of stepwise screening strategy

Test	Sensitivity % (95% CI)	Specificity % (95% CI)	PPV % (95% CI)	NPV % (95% CI)	LR+ (95% CI)	LR− (95% CI)	Overall accuracy % (95% CI)
ALT ≥ 40 and US ≥ 2 *n = 15*	33 (19–49)	98 (90–100)	93 (66–99)	65 (61–70)	18.0 (2.5–133.3)	0.7 (0.6–0.9)	70 (60–79)
ALT < 40 and US ≥ 2 *n = 44*	63 (47–77)	70 (56–81)	61 (50–72)	71 (61–79)	2.1 (1.3–3.3)	0.5 (0.4–0.8)	67 (56–76)
ULN ≤ ALT < 40 and US ≥ 2 *n = 40*	56 (40–71)	71 (78–83)	60 (48–71)	68 (59–75)	2.0 (1.2–3.2)	0.6 (0.4–0.9)	65 (54–74)
ALT ≥ ULN and US ≥ 2 *n = 30*	44 (29–60)	80 (68–90)	63 (48–76)	65 (58–72)	2.3 (1.2–4.2)	0.7 (0.5–0.9)	65 (54–74)

ULN = 22 IU/L for girls, 26 IU/L for boys

*ULN* upper limit of normal, *US* ultrasound, *ALT* alanine aminotransferase, *PPV* positive predictive value, *NPV* negative predictive value, *LR+* positive likelihood ratio, *LR−* negative likelihood ratio

## Discussion

To our knowledge, this is the first study that compares head-to-head the accuracy of ALT and US as screening tools for NAFLD in children with obesity. We show that none of the currently used screening tools for NAFLD is superior. Both ALT and US have only a mediocre accuracy (AUROC 0.74 (95% CI 0.65–0.83) and 0.70 (95% CI 0.60–0.79), respectively). When using these screening tools in practice, we consider a threshold of ALT ≥40 IU/L or US score ≥ 2 to be optimal since at these thresholds the specificity is acceptable (≥ 80%), albeit combined with a low sensitivity (44% and 51%, respectively). In practice, for reasons of costs and availability, we consider ALT the preferred screening tool in most settings.

Ideally, a screening test has a high specificity of > 95% (i.e., < 5% false positives) and a positive likelihood ratio of > 10 [28]. Our results show that for both ALT and US as screening tools for NAFLD, these criteria cannot be met unless the cutoff is put very high, resulting in an unacceptably low sensitivity. For example, using a US score ≥ 3, a specificity of 98% and a LR+ of 18 can be obtained. However, when using this cutoff, sensitivity is only 19%. In other words, 81% of the children with NAFLD will not be identified.

The results from this study underscore that physicians applying these diagnostic tests should be aware of the limited accuracy of the tests, particularly realizing that a negative screening result does not exclude NAFLD. Albeit not in the scope of this study, physicians should also be aware that ALT and US correlate not/very poorly with the presence of inflammation and fibrosis [29–31]. Thus, these tests do not provide information on the stage of NAFLD. It is important that patients are counseled on these aspects when discussing screening results.

Although no head-to-head studies comparing ALT and US exist, several pediatric studies evaluated ALT and US as screening tools for NAFLD. Comparing accuracy data from these studies is difficult as study populations and reference standards differ among studies. Two previous studies on the accuracy of US for detecting NAFLD in children with obesity using MRI as reference standard reported higher sensitivity (95% and 93%) and specificity (50% and 70%) compared with our study [9, 10]. The study population of these studies was less severely obese compared with that of our study (mean BMI *z*-score 2.5 in Pacifico et al. and 2.2 in Pozatto et al.). In addition, in these studies, a threshold of 9% MRI determined liver fat was used, without validation. This threshold is probably compatible with the detection of moderate to severe steatosis, rather than all degrees of steatosis. Likewise, in a study in children treated in a tertiary liver clinic, thus not on a population level, a US score ≥ 2 was found to have a good accuracy (sensitivity 80%, specificity 86%) for detecting moderate to severe steatosis using histology as the reference standard [11]. As previously published, in our study cohort, the accuracy of US for detecting moderate to severe steatosis is indeed higher than that for detecting all stages of steatosis [16]. Detecting mild steatosis is however equally important as detecting more severe degrees of steatosis as there is no association between the intensity of steatosis and fibrosis grade [32]. Previous studies on ALT as a screening tool in children with obesity reported a slightly better but still mediocre accuracy for detecting steatosis [9, 12–15]. Again, in these studies, children had less severe obesity compared with our study population [13] or included patients with normal BMI as well [14, 15].

As a secondary aim of this study, we evaluated whether a stepwise screening approach combining both tests would increase the accuracy of screening. None of the algorithms had an overall accuracy superior to ALT ≥40 IU/L alone. Thus, a stepwise screening strategy using ALT and US consecutively does not improve the accuracy of screening. This implies that combining both tools is not a useful strategy in the screening for NAFLD in children with obesity.

The strength of this study is that it was performed in a cohort of patients that were not selected on liver abnormalities and performed in an obesity clinic. Previous studies reporting on the accuracy of screening tools for pediatric NAFLD were frequently conducted in patients referred to hepatology departments of tertiary care hospitals, resulting in highly selected cohorts often of patients that required liver biopsy based on clinical or biochemical abnormalities. Also, all participants were well characterized and underwent a standardized liver disease assessment to exclude other causes of liver disease before inclusion. A limitation of this study is that the study population had severe obesity (mean BMI *z*-score 3.3) which might not be representative of the population in all obesity clinics. Performing a head-to-head comparison in children with less severe obesity is valuable, as the performance of US is possibly influenced by body composition. Another limitation is that the sample size of our study allowed to detect a difference of ≥ 0.15 in the AUROC curves (*α* = 0.05, *β* = 0.80). Therefore, statistical differences below 0.15 could be missed. However, we deem these differences to be not clinically relevant.

In conclusion, this study shows that none of the currently used screening tools for NAFLD is superior in children with obesity. ALT and US both have only moderate diagnostic accuracy for detecting hepatic steatosis. A stepwise screening strategy combining ALT and US does not improve the diagnostic accuracy. For practical reasons of costs and availability, ALT will be the preferred primary screening tool in most settings. Physicians should be aware of the limited accuracy of these screening tools when counseling patients, and better screening tools are needed to come to effective screening strategies for this endemic disorder.

## Abbreviations

ALT: Alanine aminotransferase
AST: Aspartate aminotransferase
AUROC: Area under the ROC curve
BMI: Body mass index
^1^H-MRS: Proton magnetic resonance spectroscopy
HOMA-IR: Homeostatic model assessment of insulin resistance score
IQR: Interquartile range
LR+: Positive likelihood ratio
LR−: Negative likelihood ratio
NAFLD: Non-alcoholic fatty liver disease
NPV: Negative predictive value
PPV: Positive predictive value
ROC: Receiver operating characteristic
SD: Standard deviation
STARD: Standards for reporting of diagnostic accuracy
ULN: Upper limit of normal
US: Ultrasound
US score: Ultrasound steatosis score
γGT: Gamma-glutamyltransferase
